# Time-Modulated Transmissive Programmable Metasurface for Low Sidelobe Beam Scanning

**DOI:** 10.34133/2022/9825903

**Published:** 2022-07-09

**Authors:** Xudong Bai, Fuli Zhang, Li Sun, Anjie Cao, Jin Zhang, Chong He, Longhai Liu, Jianquan Yao, Weiren Zhu

**Affiliations:** ^1^School of Microelectronics, Northwestern Polytechnical University, Taicang, 215400 Suzhou, China; ^2^School of Physical Science and Technology, Northwestern Polytechnical University, Xi'an 710072, China; ^3^Shanghai Institute of Satellite Engineering, Shanghai 201109, China; ^4^Department of Electronic Engineering, Shanghai Jiao Tong University, Shanghai 200240, China; ^5^College of Precision Instruments and Opto-Electronics Engineering, Institute of Laser and Optoelectronics, Tianjin University, Tianjin 300072, China

## Abstract

Programmable metasurfaces have great potential for the implementation of low-complexity and low-cost phased arrays. Due to the difficulty of multiple-bit phase control, conventional programmable metasurfaces suffer a relatively high sidelobe level (SLL). In this manuscript, a time modulation strategy is introduced in the 1-bit transmissive programmable metasurface for reducing the SLLs of the generated patterns. After the periodic time modulation, harmonics are generated in each reconfigurable unit and the phase of the first-order harmonic can be dynamically controlled by applying different modulation sequences onto the corresponding unit. Through the high-speed modulation of the real-time periodic coding sequences on the metasurface by the programmable bias circuit, the equivalent phase shift accuracy to each metasurface unit can be improved to 6-bit and thus the SLLs of the metasurface could be reduced remarkably. The proposed time-modulated strategy is verified both numerically and experimentally with a transmissive programmable metasurface, which obtains an aperture efficiency over 34% and reduced SLLs of about −20 dB. The proposed design could offer a novel approach of a programmable metasurface framework for radar detection and secure communication applications.

## 1. Introduction

Metasurfaces have been extensively researched over recent years, owing to their extraordinary talents for the fine control over electromagnetic (EM) scattering [1–5]. Based on the advantages of low profile, low cost, and high integrity, metasurfaces have shown versatile potentials in many areas, such as dynamic scattering modulation [6, 7], orbital angular momentum (OAM) beam generation [8–10], direct information transmission [11], energy harvesting [12], direction finding [13, 14], and intelligent sensing [15, 16]. Particularly, programmable metasurfaces have demonstrated their capacities in phase modulation for flexible beam forming through employing lumped components, which have exhibited significantly lower cost over conventional phased arrays using digital or analog phase shifters [17].

Programmable metamaterials were firstly proposed in 2014 to expand the concept of metamaterials through using dynamic sequences of “0” and “1” bits [18]. Thereafter, reflective or folded reflective programmable metasurfaces have been widely developed and exploited in many applications, through integrating lumped components for the dynamic alteration in metasurface unit resonant property [19–23]. Since there were serious feed blockages exhibited by the programmable reflective metasurfaces, transmissive programmable metasurfaces were proposed to improve the total radiation efficiency [24–28]. However, for all these programmable metasurfaces, only 1-bit or 2-bit phase resolutions are realized for the units, resulting in severe quantization losses of approximately 3 dB and 0.6 dB, respectively [29]. As for programmable metasurfaces with multiple-bit phase resolutions, the reconfigurable unit configurations and the corresponding control circuits would be extremely complicated for practical applications, especially in a large-scale metasurfaces. To achieve a more sophisticated EM manipulation, the time-modulated array technologies [30–32] were introduced and combined with programmable metasurfaces, and the space-time coding digital metasurfaces were thus proposed and verified to provide flexible beam steering, beam shaping, and scattering-signature control over optimized space-time coding sequences [33–35]. Many other promising applications are also conducted creatively through space-time coding digital metasurfaces, for example, implementing efficient harmonic control for far-field radiation or near-field distribution [36, 37], manipulating the nonreciprocal effects dynamically for frequency conversion [38–40], realizing smart Doppler cloaks in the microwave domain for self-adaptive anti-Doppler shift [41, 42], executing dynamic and arbitrary polarization syntheses [43, 44], extracting the accurate electromagnetic parameters of complex lossy dispersive materials [45], and more importantly, constructing the minimalist or secure wireless communication architectures through space- and frequency-division multiplexing [46–48].

For applications in radar detection, high-resolution imaging, and secure communication, low sidelobe level (SLL) is an indispensable factor for the designed radiation beams, in order to reduce the coupling interference with other components in the integrated system and minimize the possibility of being detected by the potential adversaries [49]. Due to the difficulty in implementation of multiple-bit phase control, conventional programmable metasurfaces suffer a relatively high SLL. In this manuscript, we design and fabricate a 1-bit transmissive programmable metasurface and overcome its drawback in high SLL through employing a time modulation strategy. After the periodic time modulation, the harmonics are generated in each metasurface unit, and the phase of the first-order harmonic can then be dynamically manipulated by modulation sequences applied to corresponding unit. Through the high-speed modulation of the real-time periodic coding sequences on the metasurface by the programmable bias circuit along with the high-accuracy field-programmable gate array (FPGA), the equivalent phase shift accuracy to each metasurface unit can be improved and the SLLs of the metasurface are thus reduced correspondingly. The time-modulated design strategy is verified both numerically and experimentally, and the transmissive programmable metasurface could obtain an aperture efficiency over 34% and reduced SLLs of about −20 dB, much superior to the conventional programmable metasurfaces. The proposed design could offer a novel approach of a programmable metasurface framework in low sidelobe beam scanning for radar detection and secure communication applications.

## 2. Time-Modulated Metasurface Theory

**Figure 1:**
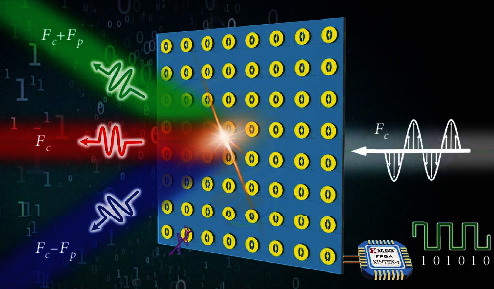
Schematic diagram of the time-modulated transmissive programmable metasurface.

The overall schematic and architecture of the time-modulated transmissive programmable metasurface is illustrated in Figure 1. A sinusoidal signal is passing through the transmissive metasurface, and harmonic beams with different frequencies are stimulated and pointing in different directions after implementing periodic time modulation. To improve the equivalent phase shift accuracy and reduce the relative SLLs, time-modulated theory is introduced and combined with the designed 1-bit transmissive programmable metasurface through the high-speed modulation of the real-time periodic coding sequences by the programmable bias circuit along with the FPGA, Xilinx Kintex-7.

Consider a 1-bit transmissive programmable metasurface with *M* × *N* elements, and the horn antenna is exploited to transmit and receive RF signals with the carrier frequency *F*_*c*_. The unit spacing along both *x* and *y* axes is *D*, and the horn antenna is located on the *z* axis with the coordinate [0, 0, −*F*] in a three-dimensional Cartesian coordinate system, where *F* is the focal length of the feed phase center.

Assume that the transmissive metasurface works on the receiving state, and the far-field incident RF signal is incident on the metasurface from the direction (*θ*, *φ*). The reconfigurable metasurface unit is composed of two patches placed on the front and back sides. The RF signals are received by the front patch and radiated out by the patches on the back, where the signal phases could be dynamically controlled through the 1-bit phase modulator using PIN diodes. The horn antenna receives the power from all the patches on the back layer, which is related to the direction of vector (*θ*_*m*,*n*_, *φ*_*m*,*n*_) and distance *l*_*m*,*n*_ from the (*m*, *n*)^th^ patch on the back side of the metasurface to the horn antenna. Here,

(1) $$\begin{array}{c} l_{m,n}=\sqrt{{\left(m-\frac{M+1}{2}\right)}^{2}D^{2}+{\left(n-\frac{N+1}{2}\right)}^{2}D^{2}+F^{2}}. \end{array}$$

Assume that the patterns of the patches on both sides are *u*_*f*_(*θ*, *φ*) and *u*_*b*_(*θ*, *φ*), respectively. The loss and phase shift of the (*m*, *n*)^th^ patch caused by the distance *l*_*m*,*n*_ are *a*(*l*_*m*,*n*_) and exp(*j*2*πl*_*m*,*n*_/*λ*), correspondingly. The array factor of the transmissive metasurface on the receiving state is thus theoretically modeled as

(2) $$\begin{array}{c} AF\left(\theta ,\varphi \right)=u_{f}\left(\theta ,\varphi \right)\times \overset{M}{\underset{m=1}{\sum }}\overset{N}{\underset{n=1}{\sum }}u_{b}\left(\theta_{m,n},\varphi_{m,n}\right)\times a\left(l_{m,n}\right)\times e^{j\left[\left(2\pi \left(l_{m,n}+d_{m,n}\right)/\lambda \right)+{\pi \omega }_{m,n}\right]}, \end{array}$$

where *ω*_*m*,*n*_ is the programmable weight and selected from the binary states {0, 1} and *d*_*m*,*n*_ is the distance from the (*m*, *n*)^th^ patch antenna on the front layer to the plane passing through the origin and perpendicular to the incident direction, which is calculated by

(3) $$\begin{array}{c} d_{m,n}=\left(m-\frac{M+1}{2}\right)D\sin\theta \cos\varphi +\left(n-\frac{N+1}{2}\right)D\sin\theta \sin\varphi . \end{array}$$

In practical, the array pattern depicted by (2) can be furtherly simplified. For example, the pattern of the patch on either side is approximately equal to cos*θ*, and *a*(*l*_*m*,*n*_) is roughly proportional to 1/*l*_*m*,*n*_^2^; thus, the array factor can be rewritten as

(4) $$\begin{array}{c} AF\left(\theta ,\varphi \right)=\overset{M}{\underset{m=1}{\sum }}\overset{N}{\underset{n=1}{\sum }}\cos\theta \times \cos\theta_{m,n}\times a\left(l_{m,n}\right)\times e^{j\left[\left(2\pi \left(l_{m,n}+d_{m,n}\right)/\lambda \right)+{\pi \omega }_{m,n}\right]}. \end{array}$$

By setting the (1, 1)^st^ unit as reference, the added periodic modulation sequence is designed as follows. In the first half of a modulation period *T*_*p*_, the phase shift is set as 0°, while in the second half, the phase shift is set as 180°. Then, the modulation sequence added to the (1, 1)^st^ unit can be described by

(5) $$\begin{array}{c} U_{1,1}\left(t\right)=\overset{\infty }{\underset{q=-\infty }{\sum }}g_{1,1}\left(t-{qT}_{p}\right),\\ g_{1,1}\left(t\right)=\left\{\begin{array}{cc} 1, & 0<t\leq \frac{T_{p}}{2},\\ -1, & \frac{T_{p}}{2}<t\leq T_{p}. \end{array}\right. \end{array}$$

The period function *U*_1,1_(*t*) can then be unfolded by the Fourier series as

(6) $$\begin{array}{c} U_{1,1}\left(t\right)=\overset{\infty }{\underset{k=-\infty }{\sum }}\alpha_{1,1,k}e^{j2\pi {kF}_{p}t}, \end{array}$$

where *F*_*p*_ = 1/*T*_*p*_ is the modulation frequency and *α*_1,1,*k*_ is the Fourier coefficient corresponding to the *k*^th^ harmonic component:

(7) $$\begin{array}{c} \alpha_{1,1,k}=\frac{1}{T_{p}}\int_{0}^{T_{p}}g_{1,1}\left(t\right)e^{-j2\pi {kF}_{p}t}dt=\left\{\begin{array}{cc} 0, & k\in \text{even},\\ \frac{-2j}{\pi k}, & k\in \text{odd}. \end{array}\right. \end{array}$$

Assume that the received signal in the (1, 1)^st^ unit is *A*_0_*e*^*j*2*πF*_*c*_*t*^. After modulation, the output can be written as

(8) $$\begin{array}{c} {Sr}_{1,1}\left(t\right)=A_{0}\overset{\infty }{\underset{k=-\infty }{\sum }}\alpha_{1,1,k}e^{j2\pi \left(F_{c}+{kF}_{p}\right)t}. \end{array}$$

Referring to (7), we see that only odd harmonic components appear after the periodic modulation and symmetric spectra with opposite harmonics exist on both sides of the carrier frequency *F*_*c*_. Among all existing harmonic components, the ±1^st^ ones are the strongest. Therefore, we choose the +1^st^ harmonic component to implement pattern synthesis. However, the −1^st^ harmonic component may cause a power waste even interference to other electronic systems. The suppression of the −1^st^ harmonic component requires a more complicated structure with 2-bit phase shift units. However, through the power suppression in other harmonic components, the gain of the time-modulated metasurface phase array can be higher than the one without modulation, owing to the effective improvement on the aperture efficiency caused by the higher phase shift accuracy.

According to (7), if a sinusoidal signal with the amplitude *A*_0_ = 1 is input to the modulation unit, the output signal would contain the +1^st^ harmonic component with the amplitude 2/*π*. The modulation sequence applied to the (*m*, *n*)^th^ unit is *U*_*m*,*n*_(*t*) = *U*_1,1_(*t* − *τ*_*m*,*n*_), where *τ*_*m*,*n*_ is the delay of *U*_*m*,*n*_(*t*) corresponding to *U*_1,1_(*t*). According to the properties of the Fourier series, *U*_*m*,*n*_(*t*) can be unfolded by

(9) $$\begin{array}{c} U_{m,n}\left(t\right)=\overset{\infty }{\underset{k=-\infty }{\sum }}\alpha_{m,n,k}e^{j2\pi F_{p}t}, \end{array}$$

(10) $$\begin{array}{c} \alpha_{m,n,k}=\alpha_{1,1,k}e^{-j2\pi {kF}_{p}\tau_{m,n}}. \end{array}$$

We see that all the time-modulated reconfigurable units would generate harmonic components at *F*_*c*_ + *kF*_*p*_. For the *k*^th^ harmonic component, the phase difference between the (*m*, *n*)^th^ unit and the (1, 1)^st^ one is decided by time delay *τ*_*m*,*n*_. Therefore, the phase shifts of the +1^st^ harmonic components for all the metasurface units can be controlled parallelly by setting different time delays *τ*_*m*,*n*_ through the FPGA.

According to (4) and (9), after time modulation, the transmissive metasurface array factor of the *k*^th^ harmonic component can be deduced as

(11) $$\begin{array}{c} AF^{\prime }\left(\theta ,\varphi \right)=\overset{M}{\underset{m=1}{\sum }}\overset{N}{\underset{n=1}{\sum }}\alpha_{m,n,k}\frac{\cos\theta \cos\theta_{m,n}}{l_{m,n}^{2}}e^{j\left(2\pi \left(l_{m,n}+d_{m,n}\right)/\lambda \right)}. \end{array}$$

By substituting (7) and (9) into (11), the array factor of the +1^st^ harmonic component can be rewritten as

(12) $$\begin{array}{c} {{AF}_{1}}^{\prime }\left(\theta ,\varphi \right)=\overset{M}{\underset{m=1}{\sum }}\overset{N}{\underset{n=1}{\sum }}\frac{-2j}{\pi }\frac{\cos\theta \cos\theta_{m,n}}{l_{m,n}^{2}}e^{j2\pi \left[\left(\left(l_{m,n}+d_{m,n}\right)/\lambda \right)-F_{p}\tau_{m,n}\right]}. \end{array}$$

Next, the phase shift accuracy for the metasurface under time modulation would be discussed thoroughly. For the FPGA device used in the programmable metasurface, there is a system clock frequency *f*_clk_, which defines the minimum time interval as 1/*f*_clk_ in all programs. That is, all the delays generated by the FPGA are an integral multiple of 1/*f*_clk_. Therefore, the phase shift resolution of the period time modulation is Δ*φ* = 2*πF*_*p*_/*f*_clk_. For example, if the system clock frequency of the FPGA is 64 MHz and the modulation frequency is 1 MHz, the equivalent phase shift accuracy under time modulation would be equal to that of a 6-bit digital phase shifter. If the required phase shift is *π*/4, the time delay of the modulation sequence should be set as 8 times of the FPGA system clock period. It is worth noting that the transmissive metasurface would operate at *F*_*c*_ + *F*_*p*_ through time modulation, and the frequency stepping among the harmonic components is 2*F*_*p*_. Therefore, in order to avoid aliasing, the bandwidth of the received signal should be less than 2*F*_*p*_.

Considering the designed 1-bit transmissive metasurface with 20 × 20 units working at 7.5 GHz, the unit spacing along both *x* and *y* axes is *λ*/2, and the feed horn antenna is placed on the *z* axis with the coordinate [0, 0, −5*λ*]. The exploited pattern synthesis method is to generate an equiphase surface perpendicular to the incidence direction. By substituting the above parameters into (4), the array pattern could be rewritten as

(13) $$\begin{array}{c} AF\left(\theta ,\varphi \right)=\overset{M}{\underset{m=1}{\sum }}\overset{N}{\underset{n=1}{\sum }}\frac{\cos\theta \cos\theta_{m,n}}{l_{m,n}^{2}}\times e^{j\left[\left(2\pi \left(l_{m,n}+d_{m,n}\right)/\lambda \right)+{\pi \omega }_{m,n}\right]}. \end{array}$$

Assume that the required phase for the (*m*, *n*)^th^ unit is *ϕ*_*m*,*n*_ in order to generate equiphase surface perpendicular to (*θ*, *φ*). For the situation without time modulation, *ω*_*m*,*n*_ in (13) is selected from the binary states {0, 1} and decided by

(14) $$\begin{array}{c} \omega_{m,n}=\left\lfloor \frac{\mathrm{mod}\left(\phi_{m,n}-\left(2\pi \left(l_{m,n}+d_{m,n}\right)/\lambda \right),2\pi \right)}{\pi }\right\rfloor , \end{array}$$

where ⌊·⌋ is the floor function.

For the situation with time modulation in our study, the system clock frequency of the FPGA is 64 MHz and the modulation frequency is 1 MHz. Therefore, the phase shift accuracy is equivalent to 6-bit at the +1^st^ harmonic component. The required phase shift *ϕ*_*m*,*n*_ is mapped to the time delay *τ*_*m*,*n*_ between the modulation sequence applied to the (*m*, *n*)^th^ unit and that of the reference unit. Thereafter, *τ*_*m*,*n*_ is generated by the control FPGA, and the number of delays over the system clock can be calculated by

(15) $$\begin{array}{c} \omega \prime_{m,n}=\left\lfloor \frac{2^{5}\times \mathrm{mod}\left(\phi_{m,n}-\left(2\pi \left(l_{m,n}+d_{m,n}\right)/\lambda \right),2\pi \right)}{\pi }\right\rfloor . \end{array}$$

As described above, in the pattern synthesis with time modulation, *ω*′_*m*,*n*_ defines the approximate ratio between *τ*_*m*,*n*_ and the system clock period.

In order to validate the effectiveness of the proposed time-modulated transmissive programmable metasurface for low SLL beam scanning, the normalized 2D patterns both with and without time modulation are calculated based on the above theory and examined further with the measured results of the fabricated transmissive programmable metasurface for four scanning directions, including (0°, 0°), (15°, 0°), (30°, 0°), and (45°, 0°). Detailed procedures along with the results are provided in the following section.

## 3. Time-Modulated Metasurface Design and Verification

**Figure 2:**
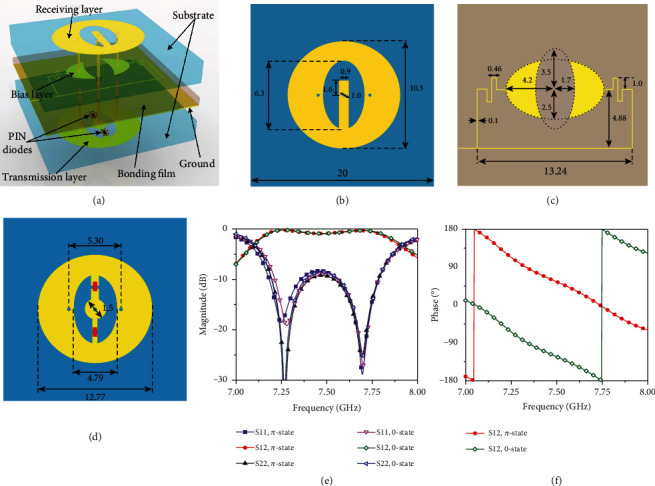
Topology and property of the transmissive programmable metasurface unit: (a) three-dimensional view, (b) receiving layer, (c) bias layer, and (d) transmission layer (item: mm). Simulated scattering coefficients for both coding states: (e) magnitude and (f) phase.

The established transmissive programmable metasurface is designed by combining a receiving patch, a transmission patch, and a 1-bit phase modulator in a single reconfigurable unit. The presented 1-bit transmissive reconfigurable unit is constructed by four metal layers along with two substrates and one bonding film, as shown in Figure 2. The two substrates are Rogers RO4003C (dielectric constant of 3.55, loss tangent of 0.0027) with a thickness of 1.524 mm, while the middle bonding film layer is Rogers 4450F (dielectric constant of 3.52, loss tangent of 0.004) with a thickness of 0.203 mm. The metal layers include, from top to bottom, the receiving layer, the bias layer, the ground, and the transmission layer. The transmission layer is designed with an elliptical patch integrated by an O-slot and two PIN diodes, while the supernatant receiving layer is with the same-size elliptical patch integrated by a U-slot, and both layers are connected at the center by a metallized via-hole through the ground for energy transferring. Besides, the receiving layer is connected with two crescent-shaped distributed capacitors in the bias layer through two symmetric distributed via-holes for biasing purposes, while the transmission layer is connected with the ground plane in a similar way. To choke the high-frequency signals ulteriorly, the bias line is designed with a very narrow linewidth, which is placed very close to the ground and integrated with two symmetric zigzag inductances along with the distributed capacitors.

The 1-bit phase resolution of the transmissive metasurface unit is obtained by integrating two PIN diodes with an antisymmetry configuration for current inversion, thus achieving the uniform amplitudes but inverse phase states for the two programmable states. The PIN diode, MACOM Flip Chip MA4FCP305 [50], is chosen to acquire lower transmission losses within the designed band and modeled by the equivalent lumped components for the two biasing states. For positive biasing, the series resistor *R*_ON_ = 2.1 *Ω* and inductor *L*_ON_ = 30 pH are adopted for the PIN diode, while for negative biasing, the series capacitance *C*_OFF_ = 0.05 pF and inductor *L*_OFF_ = 30 pH are employed for the PIN diode.

The central operation frequency of the transmissive metasurface unit is designed at 7.5 GHz. The numerical simulation is carried out with the help of the commercial software package CST Microwave Studio by using unit cell boundary conditions along with the Floquet port excitations. Figures 2(e) and 2(f) give the simulated *S* parameters of the unit in magnitude and phase for both *π* and 0 states. For the *π* state with PIN diode I on and PIN diode II off, the transmission coefficient is greater than −1 dB from 7.169 to 7.791 GHz. For the 0 state with PIN diode I off and PIN diode II on, the transmission coefficient is greater than −1 dB from 7.175 to 7.805 GHz. The phase difference of the two programmable states agrees well with 180° within the designed frequency band. Besides, the radiation characteristics of patches on both sides of the metasurface unit are also simulated, which have a gain of about 6 dBi and a beamwidth over 90°.

**Figure 3:**
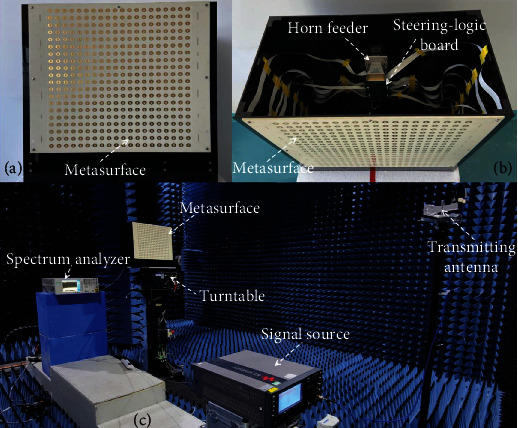
Fabricated time-modulated transmissive programmable metasurface. (a) Front view and (b) vertical view of the metasurface prototype. (c) Experimental environment and measurement setup in the microwave anechoic chamber.

To further validate the actual working performance of the proposed time-modulated transmissive programmable metasurface for low sidelobe beam scanning, the metasurface prototype with 20 × 20 units was manufactured based on the designed unit, with a unit spacing of *d* = *λ*/2 along both *x* and *y* axes. A pyramid horn feeder is positioned in the central axis of the metasurface with a focal length of *F* = 20 cm to illuminate the overall metasurface aperture, and the steering logic board is connected with the metasurface through flexible flat cables (FFCs). The metasurface along with the horn feeder and the steering logic board is all mounted onto a designed black acrylic bracket, as shown in Figure 3. Each metasurface unit is connected and modulated with the steering logic board through the bias layer network. The FPGA, Xilinx Kintex-7, is adopted as the host processing system in order to regulate the programmable signals in parallel for all 400 PIN diodes of the metasurface within the assigned clock signals, according to the optimized time-varying coding sequences. The reconfiguration speed of the proposed time-modulated transmissive programmable metasurface depends critically on the minimum switching speed of the PIN diode employed, which is about 20 nanoseconds according to the data sheet [50].

The experimental environment and measurement setup in the microwave anechoic chamber are also presented in Figure 3. A horn antenna transmits the single-frequency signal at 7.5 GHz stimulated by the signal source from the far field. The transmissive programmable metasurface is fastened on an electronic-controlled turntable, and the power patterns were recorded by the spectrum analyzer. Firstly, the programmable metasurface is operating with time modulation. The main clock frequency of the FPGA on the control board is set as 64 MHz with modulation frequency of 1 MHz, resulting in an equivalent phase shift accuracy of 6 bits. The pattern synthesis method is to generate an equiphase surface perpendicular to the desired beam direction, and the weight *ω*′_*m*,*n*_ is calculated by (15) and then translated to the delay of the modulation sequence compared to the reference in programs. As for the situation without time modulation, the weight *ω*_*m*,*n*_ is calculated by (14).

**Figure 4:**
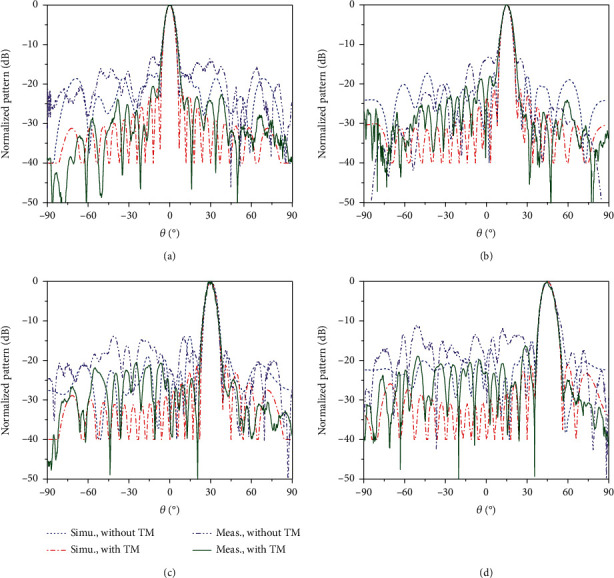
Measured and simulated normalized 2D radiation patterns both with and without time modulation for four scanning directions: (a) theta = 0°, (b) theta = +15°, (c) theta = +30°, and (d) theta = +45°.

The measured normalized 2D radiation patterns both with and without time modulation are plotted and compared with the calculated results based on the above time-modulated metasurface theory, as shown in Figure 4. It is observed that good-shaped directional pencil beams for the four scanning directions, including (0°, 0°), (15°, 0°), (30°, 0°), and (45°, 0°), are obtained with very high pointing stability. The main lobes of the scanning beams with and without time modulation are nearly the same, while the SLLs would decrease remarkably by using time modulation. The measured SLLs are lower than −12 dB for the programmable metasurface without time modulation, while that with time modulation would be lower than −20 dB for the scanning beams, reaching a −10 dB decrease on average. The measured results are basically coincident with the calculated predictions, and the relatively higher SLLs of the measured patterns are mainly caused by the assembly error along with the estimation error of the metasurface patch pattern. The beamwidth of the main lobes would be broadened as the scanning angle increases and the SLLs of the metasurface also have a little increase but still remain lower than −20 dB for the scanning beams through time modulation. The maximum measured gain of the metasurface with time modulation is over 26.31 dB with an aperture efficiency in excess of 34%, while a decreased aperture efficiency lower than 30% would be obtained for the programmable metasurface without time modulation. The aperture efficiency is improved because of the high phase accuracy created with time modulation, along with a higher energy ratio achieved for the main lobes through sidelobe suppression.

In order to further highlight the intrinsic advantage of the proposed design, a comparison of the overall performance for the proposed time-modulated transmissive programmable metasurface with some representative published works is tabulated in Table 1. Owing to the equivalent 6-bit phase accuracy created through time modulation, an increased aperture efficiency over 34% along with the reduced SLLs of about −20 dB could be obtained, which has definite improvements when compared with the conventional programmable metasurfaces without time modulation.

## 4. Conclusion

In summary, we have presented a time modulation strategy for increasing the phase shift accuracy of a 1-bit transmissive programmable metasurface and decreasing the SLLs of the scanning beams. Numerical simulations were provided, and a 20 × 20-unit transmissive programmable metasurface was fabricated and measured to validate the effectiveness of the proposed strategy. Through the high-speed modulation of the real-time periodic coding sequences on the metasurface by the programmable bias circuit, an improved 6-bit phase shift accuracy was achieved at the +1^st^ harmonic component for the time-modulated metasurface. We demonstrated that the aperture efficiency is improved along with the SLLs reduced owing to the high phase accuracy created through time modulation. The time-modulated transmissive programmable metasurface could obtain a measured gain over 26.31 dB along with an aperture efficiency over 34% and reduced SLLs of about −20 dB. The proposed design could offer a novel approach of a programmable metasurface framework in low sidelobe beam scanning for prospective radar detection and secure communication applications.

## Figures and Tables

**Table 1:** Comparison for programmable metasurfaces realized in literatures.

Mode of operation	Phase resolution	Gain	SLL	Aperture efficiency
Reflection [19]	1-bit	22.5 dB	-10.0 dB	22.2%
Folded reflection [21]	1-bit	22.7 dB	-8.0 dB	14.8%
Transmission [26]	1-bit	27.0 dB	-11.0 dB	17.5%
Time-modulated transmission (this work)	Equivalent 6-bit	26.3 dB	-20.0 dB	34.0%

## Data Availability

The data used to support the findings of this study are available within the article and the supplementary materials. Raw data are available from the corresponding authors upon reasonable request.

## Supplementary Materials

Supplementary Materials Supplementary Materials Figure S1: simulated 3D polar power patterns with and without time modulation for scanning direction (20°, 0°). (a) Without time modulation and (b) with time modulation. Figure S2: measured power patterns with equivalent 2-bit, 4-bit, and 6-bit phase shift accuracy. (Supplementary Materials).

## References

[1] Yu N., Genevet P., Kats M. A. (2011). Light propagation with phase discontinuities: generalized laws of reflection and refraction. *Science*.

[2] Mueller J. P. B., Rubin N. A., Devlin R. C., Groever B., Capasso F. (2017). Metasurface polarization optics: independent phase control of arbitrary orthogonal states of polarization. *Physical Review Letters*.

[3] Li A., Singh S., Sievenpiper D. (2018). Metasurfaces and their applications. *Nanophotonics*.

[4] Arbabi E., Kamali S. M., Arbabi A., Faraon A. (2019). Vectorial holograms with a dielectric metasurface: ultimate polarization pattern generation. *ACS Photonics*.

[5] Lassaline N., Brechbühler R., Vonk S. J. W. (2020). Optical fourier surfaces. *Nature*.

[6] Li Y., Lin J., Guo H., Sun W., Xiao S., Zhou L. (2020). A tunable metasurface with switchable functionalities: from perfect transparency to perfect absorption. *Advanced Optical Materials*.

[7] Song X., Bai X., Zhu W. (2022). Reconfigurable metasurface for nearly full-range and continuous modulation of reflection, transmission, and absorption. *ACS Applied Electronic Materials*.

[8] Akram M. R., Ding G., Chen K., Feng Y., Zhu W. (2020). Ultrathin single layer metasurfaces with ultra-wideband operation for both transmission and reflection. *Advanced Materials*.

[9] Liu B., He Y., Wong S. W., Li Y. (2021). Multifunctional vortex beam generation by a dynamic reflective metasurface. *Advanced Optical Materials*.

[10] Bai X., Zhang F., Sun L. (2022). Dynamic millimeter-wave OAM beam generation through programmable metasurface. *Nanophotonics*.

[11] Cui T. J., Liu S., Bai G. D., Ma Q. (2019). Direct transmission of digital message via programmable coding metasurface. *Research*.

[12] Han J., Li L., Ma X. (2022). Adaptively smart wireless power transfer using 2-bit programmable metasurface. *IEEE Transactions on Industrial Electronics*.

[13] Hoang T. V., Sharma R., Fusco V., Yurduseven O. (2021). Single-pixel compressive direction of arrival estimation using programmable metasurface apertures. *Proceedings of SPIE - The International Society for Optical Engineering*.

[14] Wang J. W., Huang Z. A., Xiao Q. (2022). High-precision direction-of-arrival estimations using digital programmable metasurface. *Advanced Intelligent Systems*.

[15] Li L., Shuang Y., Ma Q. (2019). Intelligent metasurface imager and recognizer. *Light: science & applications*.

[16] Wang J., Kühne J., Karamanos T., Rockstuhl C., Maier S. A., Tittl A. (2021). All-dielectric crescent metasurface sensor driven by bound states in the continuum. *Advanced Functional Materials*.

[17] Taghvaee H., Cabellos-Aparicio A., Georgiou J., Abadal S. (2020). Error analysis of programmable metasurfaces for beam steering. *IEEE Journal on Emerging and Selected Topics in Circuits and Systems*.

[18] Cui T. J., Qi M. Q., Wan X., Zhao J., Cheng Q. (2014). Coding metamaterials, digital metamaterials and programmable metamaterials. *Light: science & applications*.

[19] Xu H., Xu S., Yang F., Li M. (2020). Design and experiment of a dual-band 1 bit reconfigurable reflectarray antenna with independent large-angle beam scanning capability. *IEEE Antennas and Wireless Propagation Letters*.

[20] Kiani M., Tayarani M., Momeni A., Rajabalipanah H., Abdolali A. (2020). Self-biased tri-state power-multiplexed digital metasurface operating at microwave frequencies. *Optics Express*.

[21] Wang Z., Ge Y., Pu J. (2020). 1 bit electronically reconfigurable folded reflectarray antenna based on p-i-n diodes for wide-angle beam-scanning applications. *IEEE Transactions on Antennas and Propagation*.

[22] Shabanpour J., Beyraghi S., Ghorbani F., Oraizi H. (2021). Implementation of conformal digital metasurfaces for THz polarimetric sensing. *OSA Continuum*.

[23] Frazier B. W., Antonsen T. M., Anlage S. M., Ott E. (2022). Deep-learning estimation of complex reverberant wave fields with a programmable metasurface. *Physical Review Applied*.

[24] Li H., Li Y. B., Chen G. (2021). High-resolution near-field imaging and far-field sensing using a transmissive programmable metasurface. *Advanced Materials Technologies*.

[25] Taravati S., Eleftheriades G. V. (2021). Programmable nonreciprocal meta-prism. *Scientific Reports*.

[26] Pham T. K., Guang L., González-Ovejero D., Sauleau R. (2021). Dual-band transmitarray with low scan loss for satcom applications. *IEEE Transactions on Antennas and Propagation*.

[27] Bai X., Kong F., Sun Y. (2020). High-efficiency transmissive programmable metasurface for multimode OAM generation. *Advanced Optical Materials*.

[28] Huang C., Pan W., Luo X. (2016). Low-Loss circularly polarized transmitarray for beam steering application. *IEEE Transactions on Antennas and Propagation*.

[29] Yang H., Yang F., Xu S. (2017). A study of phase quantization effects for reconfigurable reflectarray antennas. *IEEE Antennas and Wireless Propagation Letters*.

[30] He C., Liang X., Li Z., Geng J., Jin R. (2015). Direction finding by time-modulated array with harmonic characteristic analysis. *IEEE Antennas and Wireless Propagation Letters*.

[31] Chen J., Liang X., He C. (2017). Efficiency improvement of time modulated array with reconfigurable power divider/combiner. *IEEE Transactions on Antennas and Propagation*.

[32] He C., Cao A., Chen J. (2018). Direction finding by time-modulated linear array. *IEEE Transactions on Antennas and Propagation*.

[33] Zhang L., Chen X. Q., Liu S. (2018). Space-time-coding digital metasurfaces. *Nature Communications*.

[34] Zhang L., Cui T. J. (2021). Space-time-coding digital metasurfaces: principles and applications. *Research*.

[35] Taravati S., Eleftheriades G. V. (2022). Microwave space-time-modulated metasurfaces. *ACS Photonics*.

[36] Zhang C., Yang J., Yang L. X. (2020). Convolution operations on time-domain digital coding metasurface for beam manipulations of harmonics. *Nanophotonics*.

[37] Yang J., Ke J. C., Chen M. (2021). Control of the harmonic near-field distributions by an active metasurface loaded with pin diodes. *Photonics Research*.

[38] Zhang L., Chen X. Q., Shao R. W. (2019). Breaking reciprocity with space-time-coding digital metasurfaces. *Advanced Materials*.

[39] Liu M., Powell D. A., Zarate Y., Shadrivov I. V. (2018). Huygens’ metadevices for parametric waves. *Physical Review X*.

[40] Wang X., Han J., Tian S., Xia D., Li L., Cui T. J. (2022). Amplification and manipulation of nonlinear electromagnetic waves and enhanced Nonreciprocity using transmissive space-time-coding metasurface. *Advanced Science*.

[41] Ramaccia D., Sounas D. L., Alù A., Toscano A., Bilotti F. (2020). Phase-induced frequency conversion and Doppler effect with time-modulated metasurfaces. *IEEE Transactions on Antennas and Propagation*.

[42] Zhang X. G., Sun Y. L., Yu Q. (2021). Smart Doppler cloak operating in broad band and full polarizations. *Advanced Materials*.

[43] Ke J. C., Dai J. Y., Chen M. Z. (2021). Linear and nonlinear polarization syntheses and their programmable controls based on anisotropic time-domain digital coding metasurface. *Small structures*.

[44] Hu Q., Chen K., Zhang N. (2022). Arbitrary and dynamic Poincaré sphere polarization converter with a time-varying metasurface. *Advanced Optical Materials*.

[45] Ghasemi S., Rajabalipanah H., Tayarani M., Abdolali A., Baharian M. (2021). Time-modulated metasurface-assisted measurements. *Advanced Optical Materials*.

[46] Zhao J., Yang X., Dai J. Y. (2019). Programmable time-domain digital-coding metasurface for non-linear harmonic manipulation and new wireless communication systems. *National Science Review*.

[47] Zhang L., Chen M. Z., Tang W. (2021). A wireless communication scheme based on space- and frequency-division multiplexing using digital metasurfaces. *Nature Electronics*.

[48] Sedeh H. B., Salary M. M., Mosallaei H. (2022). Active multiple access secure communication enabled by graphene-based time-modulated metasurfaces. *IEEE Transactions on Antennas and Propagation*.

[49] Panahi M. A., Yousefi L., Shahabadi M. (2015). Highly directive hybrid plasmonic leaky-wave optical antenna with controlled side-lobe level. *Journal of Lightwave Technology*.

[50] MACOM MACOM MA4FCP305 solderable AlGaAs flip chip PIN diodes. https://www.macom.com/products/product-detail/MA4FCP305.

