# Supplementary material for: Time-Modulated Transmissive Programmable Metasurface for Low Sidelobe Beam Scanning
Source: Research (Wash D C). 2022 Jul 9;2022:9825903. doi: 10.34133/2022/9825903 (PMC9297726; doi:10.34133/2022/9825903)
Supplement: Supplementary Materials — Figure S1: simulated 3D polar power patterns with and without time modulation for scanning direction (20°, 0°). (a) Without time modulation and (b) with time modulation. Figure S2: measured power patterns with equivalent 2-bit, 4-bit, and 6-bit phase shift accuracy. (Supplementary Materials). [file 9825903.f1.docx]

**Supplementary Material for**

Time-Modulated Transmissive Programmable Metasurface for Low Side-Lobe Beam Scanning

**Authors**

Xudong Bai^1^, Fuli Zhang^1,2^*, Li Sun^1^, Anjie Cao^3^, Jin Zhang^4^, Chong He^4^*, Longhai Liu^5^, Jianquan Yao^5^, and Weiren Zhu^4^*

**Affiliations**

^1^ School of Microelectronics, Northwestern Polytechnical University, Taicang 215400, Suzhou, China

^2^ School of Physical Science and Technology, Northwestern Polytechnical University, Xi'an 710072, China

^3^ Shanghai Institute of Satellite Engineering, Shanghai 201109, China

^4^ Department of Electronic Engineering, Shanghai Jiao Tong University, Shanghai 200240, China

^5^ College of Precision Instruments and Opto-Electronics Engineering, Institute of Laser and Optoelectronics, Tianjin University, Tianjin 300072, China

* Correspondence should be addressed to Fuli Zhang; [fuli.zhang@nwpu.edu.cn](mailto:fuli.zhang@nwpu.edu.cn), Chong He; hechong@sjtu.edu.cn and Weiren Zhu; weiren.zhu@sjtu.edu.cn

**Supplementary Note 1: Simulated 3D polar power patterns**

Considering the designed 1-bit transmissive metasurface with $20\times20$ units working at 7.5 GHz, the unit spacing along both *x* and *y* axes is $\lambda/2$. The system clock frequency of the FPGA is set as 64 MHz and the modulation frequency is 1 MHz. Therefore, the phase shift accuracy is equivalent to 6-bit at the $+1^{\text{st}}$ harmonic component. The pattern synthesis method is employed to generate equiphase surface perpendicular to the desired beam direction, and the weight $\omega'_{m,n}$ is calculated by (14), and then translated to the delay of modulation sequence compared to the reference in programs. As for the situation without time modulation, the weight $\omega_{m,n}$ is calculated by (13). The simulated 3D polar power patterns with and without time modulation for scanning direction $(20^{\circ},0^{\circ})$ are plotted based on the time-modulated metasurface theory, as shown in **Figure S1**, and the SLLs would decrease remarkably through using time modulation.


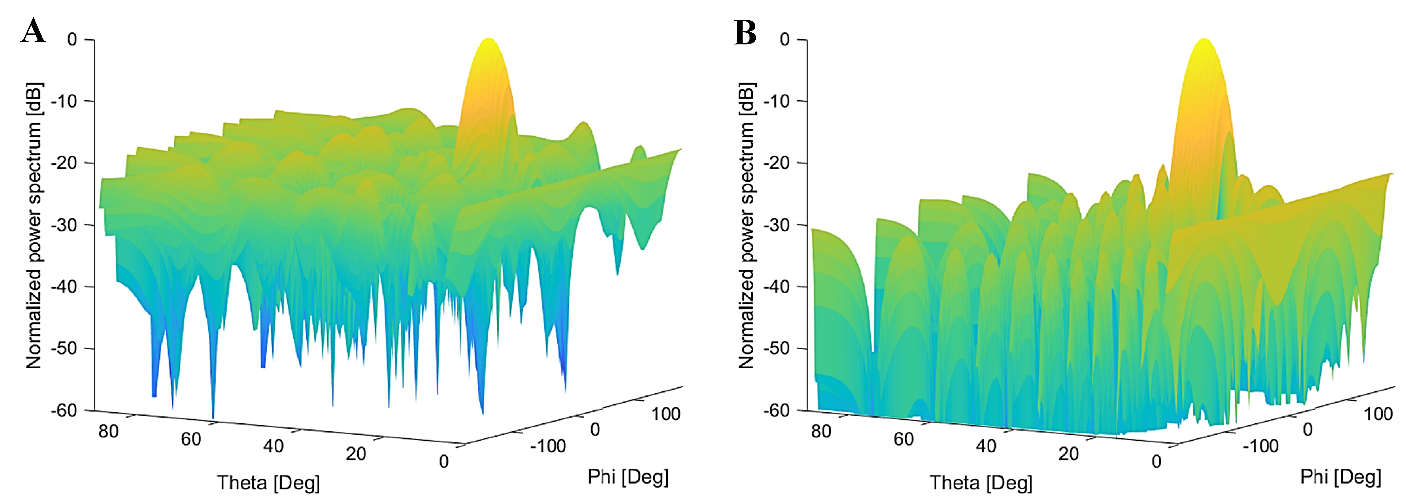


***Figure S1. Simulated 3D polar power patterns with and without time modulation for scanning direction (20°, 0°).*** *(a) Without time modulation, (b) with time modulation.*

**Supplementary Note 2: Measured power patterns for multiple-bit time modulation**

According to the detailed analysis of the time-modulated metasurface theory, the phase shift accuracy can be reconfigured by setting different system clock frequencies and modulation frequencies through dynamic time modulation. To further validate the effectiveness of the proposed design, the power patterns of the time-modulated metasurface under different equivalent phase shift accuracy are measured and compared, as shown in **Figure S2.** As the equivalent phase shift accuracy increases from 2-bit to 6-bit, the SLLs have experienced a significant decline.





***Figure S2. Measured power patterns with equivalent 2-bit, 4-bit and 6-bit phase shift accuracy.***
